# Cardiac Tamponade as a Manifestation of Clear Cell Renal Cell Carcinoma

**DOI:** 10.7759/cureus.15658

**Published:** 2021-06-15

**Authors:** María Teresa Calzada, Ignacio Boira, Eusebi Chiner, Violeta Esteban, José Norberto Sancho

**Affiliations:** 1 Cardiology, Hospital Universitario San Juan Alicante, San Juan Alicante, ESP; 2 Pulmonology, Hospital Universitario San Juan Alicante, San Juan Alicante, ESP

**Keywords:** pleural effusion, tamponade, pericardial effusion, clear cell carcinoma, pericardiocentesis

## Abstract

Some solid cancers (such as lung, breast, and esophageal cancer, and melanoma) can lead to pericardial effusion by metastatic spread, potentially provoking hemodynamic instability. Detection by echocardiography is therefore essential.

Pericardiocentesis can help restore cardiac function and provide fluid for establishing an etiology through cytological, microbiological, and cellularity analysis.

A 60-year-old woman with metabolic syndrome and obesity hypoventilation syndrome presented to the emergency department with dyspnea at rest. A chest X-ray showed cardiomegaly and massive left pleural effusion. Ultrasound findings were pericardial effusion with signs of cardiac tamponade. We performed pericardiocentesis, extracting 1000 mL of exudate, and thoracentesis, which confirmed the diagnosis of lymphocytic exudative effusion. A CAT (computerized tomography) scan of the chest, abdomen, and pelvis revealed a left kidney mass. A biopsy of the mass confirmed the diagnosis of clear cell renal cell carcinoma and a pleural biopsy revealed metastatic involvement.

This report describes a rare presentation of cardiac tamponade due to clear cell renal cell carcinoma and discusses the pathogenesis, mechanisms, and prognosis of this condition.

## Introduction

Cardiac tamponade due to neoplastic pericardial effusion is rarely associated with solid cancers (lung, breast, and esophageal cancer, and melanoma) [1]. Clear cell carcinoma can develop in multiple tissue types, including the kidney, uterus, gastrointestinal tract, or ovary. There are few published cases of cardiac tamponade secondary to all types of clear cell carcinoma [2]. The most common manifestations of renal cell clear cell carcinoma include non-metastatic hypertransaminasemia (Stauffer syndrome), varicocele, and bone metastasis.

We present the case of a patient with recurrent pericardial effusion with signs of tamponade, and recurrent pleural effusion, in the early stages of clear cell renal cell carcinoma.

## Case presentation

The patient was a 60-year-old woman, nonsmoker, with morbid obesity, hypertension, type 1 diabetes, dyslipidemia, poorly controlled asthma, diabetic nephropathy, and chronic anemia. She was on long-term home oxygen therapy and bi-level positive airway pressure ventilation for chronic type 2 respiratory failure and obesity hypoventilation syndrome.

**Figure 1:**
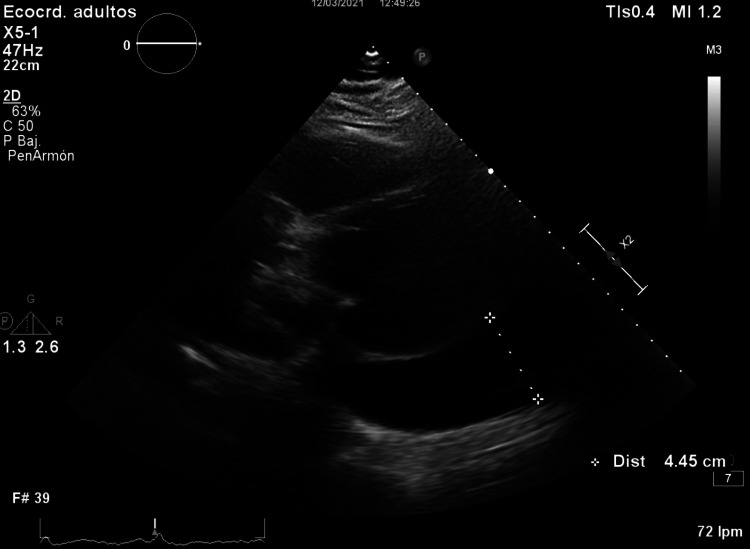
Ultrasound image showing severe pleuropericardial effusion.

She presented to the emergency department after her usual dyspnea worsened, persisting when she was at rest, with no fever, expectoration, chest pain, syncope, or constitutional symptoms. Physical examination showed a body mass index of 46 kg/m2, jugular vein distention, imperceptible heart sounds, and reduced vesicular breath sounds on the left side of the chest. A chest X-ray showed cardiomegaly and massive left pleural effusion. Blood test results were hemoglobin (Hb) 8.3 g/dL, mean corpuscular volume (MCV) 73 fL, lactate dehydrogenase (LDH) 525 U/L, D-dimer 5400 ng/mL, albumin 3 g/dL, C-reactive protein (CRP) 3 mg/dL. Arterial blood gas results (fraction of inspired oxygen [FiO2] 0.21) were pH 7.36, partial pressure of carbon dioxide (PCO2) 49 mmHg, partial pressure of oxygen (PO2) 67 mmHg, bicarbonate (HCO3) 27 mmol/L, and lactate 0.7 mmol/L. An ultrasound scan showed pericardial effusion with signs of cardiac tamponade (Figure 1).

We, therefore, performed pericardiocentesis, removing 1000 mL of amber-colored fluid with a fluid protein to serum protein ratio of 0.66 and a fluid LDH to serum LDH ratio of 0.72. These findings confirmed the diagnosis of exudative effusion. Cytology and culture results were negative. Autoimmune test results were also negative, and biochemical analysis revealed a CA-125 level of 47 U/mL. A ventilation/perfusion scan ruled out pulmonary thromboembolism. Thoracentesis produced bloody fluid with predominantly mononuclear cells, a fluid protein to serum protein ratio of 0.55, and a fluid LDH to serum LDH ratio of 1.5, confirming lymphocytic exudative effusion. Cytological and microbiological results were negative.

**Figure 2:**
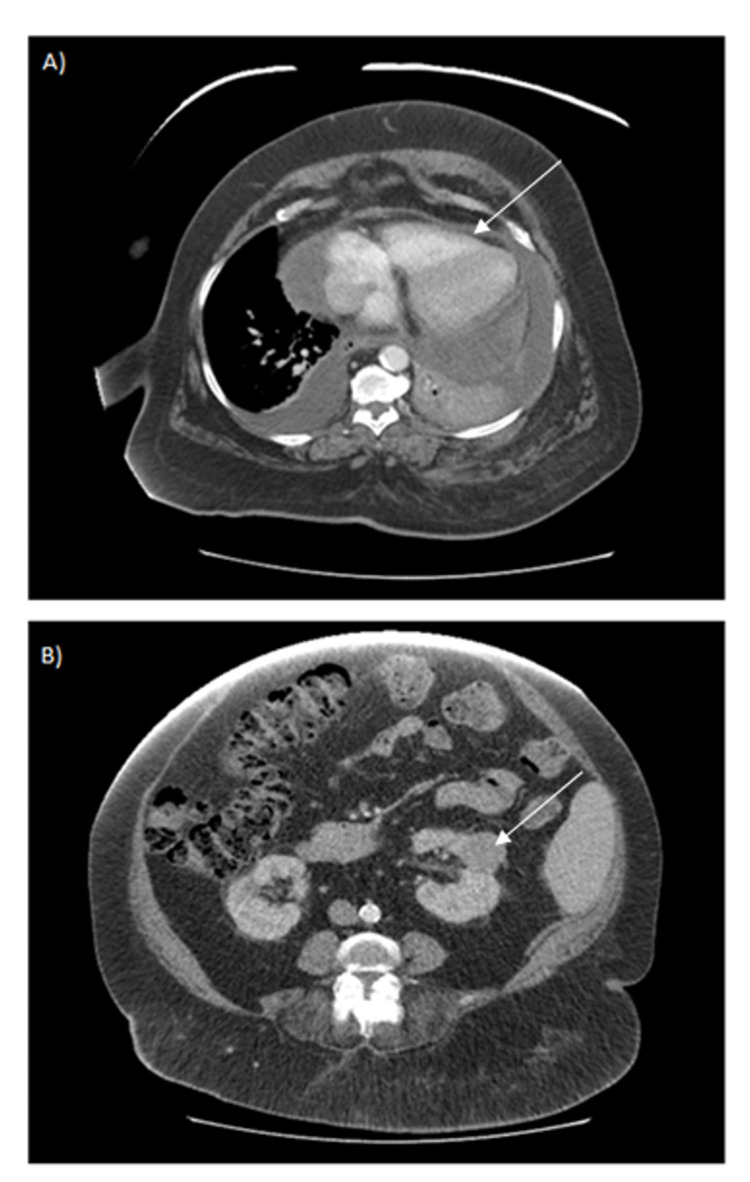
A) Chest CT scan showing a large pericardial effusion of heterogeneous density, and bilateral, left-predominant pleural effusion. B) Abdominal CT scan showing a solid mass in the interpolar cortex of the left kidney, measuring approximately 3.5 cm in diameter.

These findings suggested tumoral etiology, and we, therefore, ordered a CAT scan of the chest, abdomen, and pelvis. This technique revealed a large pericardial effusion (Figure 2A); pathological right hilar, right paratracheal and prevascular lymphadenopathy; bilateral pleural effusion; splenomegaly; and a left kidney mass (Figure 2B).

In view of the recurrent pericardial effusion, we performed a pericardial window.

In addition, pleural drainage was inserted due to recurrent pleural effusion and pleurodesis was administered with 500 mg of intrapleural doxycycline. Because of the patient´s high risk, only palliative treatment was considered.

**Figure 3:**
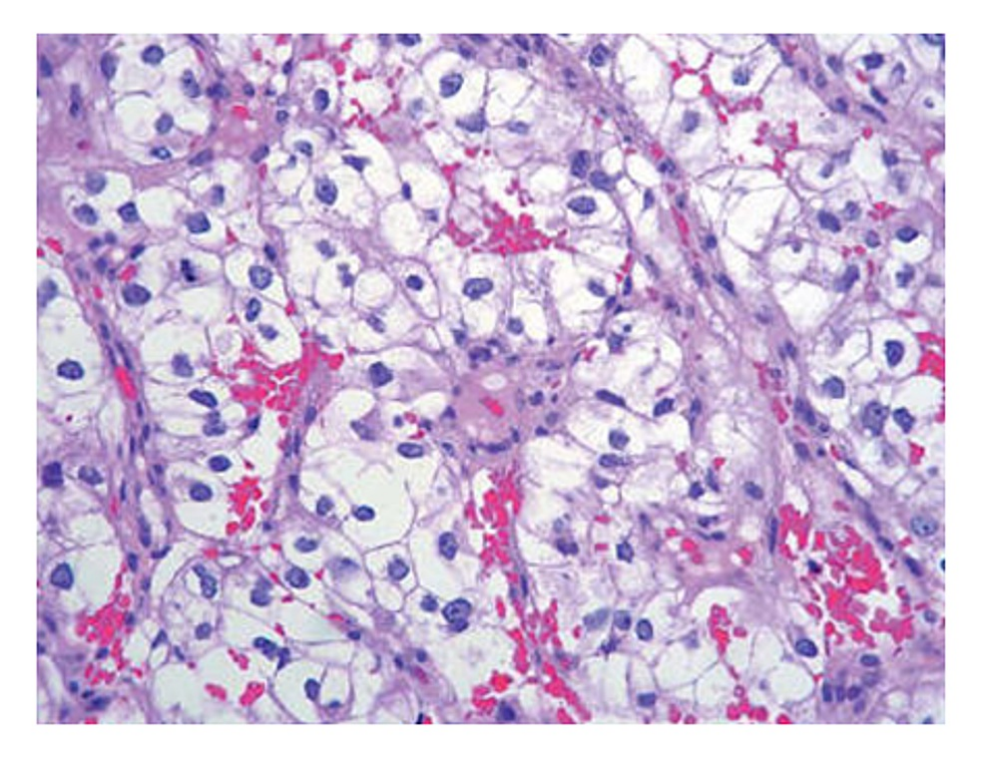
Biopsy of the clear cell renal cell carcinoma. Hematoxylin and eosin staining; magnification 400x; alveolar pattern; cells with clear cytoplasm and moderately pleomorphic nuclei.

A biopsy of the kidney lesion (Figure 3) confirmed the diagnosis of clear cell renal cell carcinoma, and a pleural biopsy revealed metastatic involvement.

## Discussion

Cardiac tamponade is a clinical entity caused by an abnormal accumulation of fluid in the pericardial sac, resulting in increased intracardiac pressures that prevent normal cardiac filling and reduce cardiac output, sometimes to extremely low levels. Rapid fluid accumulation can overwhelm the compensatory reflexes that maintain cardiac output, potentially leading to cardiogenic shock. Clinically, this translates to tachypnea, tachycardia, and hemodynamic instability with high systemic venous pressure, accompanied by signs such as increased jugular vein pressure on inspiration (Kussmaul's sign) and abnormally reduced systolic pressure on inspiration (pulsus paradoxus) [1].

Cardiac tamponade is a manifestation frequently associated with malignancy. The most common causative cancers are solid and hematologic cancers, while primary neoplasms are rare. In cancer patients, pericardial effusion is caused by the direct or metastatic spread of the primary process or as a complication of antineoplastic therapies. Pericardial effusion is sometimes the first manifestation of the disease, as in our case. For this reason, malignant causes must be ruled out in all cases of pericardial effusion presenting as tamponade, and in all cases of rapidly increasing or recurrent pericardial effusion [2].

The prevalence of cardiac tamponade in neoplastic pericardial effusion is poorly understood. In a series of 96 cases of cardiac tamponade, 52% were secondary to neoplasms, with no evidence of renal origin in any case. Primary neoplasms include solid cancers such as lung and breast cancer, esophageal carcinoma, melanoma, and thymic carcinoma; and in rare cases, germ cell, renal, bladder, Merkel cell, or blood cancers [3].

Lung carcinoma is the primary tumor with the greatest pericardial involvement, accounting for up to 50% of cases of malignant cardiac tamponade. This is because it can spread to the mediastinal lymph nodes, from where it spreads retrogradely to the epicardial lymphatic plexus, increasing the volume of pericardial fluid [4].

The association between clear cell renal cell carcinoma [5] and cardiac tamponade, described in very few publications, is caused by the intravascular spread of the tumor (through the renal vein and inferior vena cava), leading in some cases to inferior vena cava thrombosis and right ventricular dysfunction. Cardiac involvement [6] without intravascular spread has been described in only two publications. According to the literature, clear cell renal cell carcinoma is found to have spread to the right atrium in fewer than 1% of cases at the time of nephrectomy. Post-mortem studies have revealed cardiac metastases in 11% of patients who die from renal clear cell carcinoma [7].

Metastasis can also occur by systemic spread through the intrathoracic lymphatic system, especially in the presence of disseminated disease and lung metastases.

Intrapericardial therapy with cisplatin or other agents may help to prevent recurring neoplastic pericardial effusion due to lung cancer and has also shown efficacy in effusion secondary to renal cell carcinoma when combined with oral colchicine [8]. Nevertheless, tumoral pericardial effusion has a poor prognosis, as it is considered an expression of widespread disease.

## Conclusions

Cardiac tamponade is a manifestation frequently associated with malignancy. The association between clear cell renal cell carcinoma and cardiac tamponade is described in very few publications. We present the case of a patient with recurrent pericardial effusion with signs of tamponade, and recurrent pleural effusion, in the early stages of clear cell renal cell carcinoma.
